# Comment on Kerro Dego et al. Evaluation of *Streptococcus uberis* Surface Proteins as Vaccine Antigens to Control *S. uberis* Mastitis in Dairy Cows. *Vaccines* 2021, *9*, 868

**DOI:** 10.3390/vaccines13020141

**Published:** 2025-01-29

**Authors:** Rosa Collado, Carlos Montbrau, David Sabaté, Antoni Prenafeta

**Affiliations:** Hipra Scientific, S.L.U., Avda. La Selva 135, 17170 Amer, Spain; carlos.montbrau@hipra.com (C.M.); antoni.prenafeta@hipra.com (A.P.)

**Keywords:** mastitis, vaccine, efficacy

A recent publication by Kerro Dego et al. [1] mentioned in its abstract the non-existence of effective vaccines against *Streptococcus uberis* mastitis in dairy cows. We cannot agree with this statement and wish to add a few comments. This statement is at least biased in terms of its definition of efficacy. As is well known, the efficacy of a vaccine is determined by conducting preclinical and clinical trials to determine the reduction in the different parameters associated with an infection [2]. The vast majority of commercial veterinary vaccines induce a clear reduction in the different clinical parameters of the disease but are not able to prevent infection in 100% of those vaccinated. This fact does not mean that vaccines are not effective.

Taking into account the fact that the widespread prophylactic use of antibiotics is no longer sustainable, and considering its importance as a common mastitis control practice, vaccination will be a sustainable procedure to minimize the incidence of clinical and subclinical mastitis [3]. Unfortunately, nowadays, we agree that vaccination alone cannot prevent mastitis. But the utilization of vaccines in combination with other infection control measures, such as excellent milking procedures, treatment, segregation, and culling of known infected cattle, could lead to a substantial reduction in the incidence and duration of intramammary infections [4], as previously described with the use of Startvac^®^ (*Staphylococcus aureus* and *Escherichia coli* vaccine from Laboratorios Hipra, S.A); or UBAC^®^ (*S. uberis* vaccine from Laboratorios Hipra, S.A) [5,6]. Therefore, describing vaccines as non-effective could lead to misperceptions.

Additionally, in Europe, any veterinary vaccine registered must comply with the European Pharmacopeia. These guidelines specify the need to perform controlled experimental studies and field-based studies to establish the label claims for a product [2]. This means that only those claims that have been demonstrated in preclinical and clinical trials can be included in the product label. Therefore, contrary to what is stated by Kerro Dego et al. [1], UBAC^®^ has been well characterized in controlled experimental studies and field-based studies to confirm its label claims [7]. As described by Collado et al. [5], UBAC^®^ vaccine has been proven to be efficacious in a controlled experimental study. In this study, results showed that all challenged quarters developed clinical mastitis. Nevertheless, vaccination significantly reduced the clinical signs of mastitis, bacterial count, rectal temperature, and daily milk yield losses after the intramammary infection, and significantly increased the number of quarters with no bacterial isolation and SCC < 200,000 cells/mL at the end of the study. With regard to field-based studies, Puig et al. [6] described the efficacy of the UBAC^®^ vaccine under field conditions, where 401 vaccinated and 380 control cows in six herds were enrolled. The study showed a significant reduction of 52.5% in the incidence of *S. uberis* clinical mastitis and SCC in animals with *S. uberis* subclinical mastitis (997 vs. 3274 × 10^3^ cells/mL). In addition, vaccination with UBAC^®^ reduced milk production losses amongst animals with *S. uberis* subclinical mastitis (39.3 vs. 36.6 L/d).

Finally, with regard to the type of immunological response induced by UBAC^®^, it must be clarified that Collado et al. [5] showed clear data on the adaptive (humoral) immune response generated through vaccination with UBAC^®^, contrary to what is stated by Kerro Dego et al. [1]. Specifically, data on the immunoglobulin G2 response against lipoteichoic acid (LTA) in sera and milk samples from the vaccinated and control groups were presented in [5]. These data demonstrate that cows vaccinated with UBAC^®^ developed a specific humoral immune response against *S. uberis* LTA. The presence of these antibodies in milk, as explained by Collado et al. [5], could interfere with the binding of the bacteria to the host epithelial cells, as suggested by Czabanska et al. [8], when an intramammary infection occurs, preventing the subsequent biofilm formation. This could lead to a reduced *S. uberis* colonization rate of the mammary gland, which has been suggested as the mechanism of protection in other experimental vaccines [9,10,11]. Further studies will be needed in order to prove this hypothesis; however, the fact that UBAC^®^ induces a humoral immune response against *S. uberis* LTA is not in doubt.
